# CYPD limits HR^+^ mammary carcinogenesis in mice

**DOI:** 10.1038/s41420-025-02555-0

**Published:** 2025-06-10

**Authors:** Aitziber Buqué, Manuel Beltrán-Visiedo, Ai Sato, Claudia Galassi, Giulia Petroni, Lorenzo Galluzzi

**Affiliations:** 1 Department of Radiation Oncology, Weill Cornell Medical College, New York, NY USA; 2 Cancer Signaling and Microenvironment Program, Fox Chase Cancer Center, Philadelphia, PA USA; 3 Department of Pharmacology, Weill Cornell Medical College, New York, NY USA; 4 Department of Experimental and Clinical Medicine, University of Florence, Florence, Italy

**Keywords:** Breast cancer, Cell death

## Abstract

Mitochondrial permeability transition (MPT)-driven necrosis and necroptosis are regulated variants of cell death that can drive inflammation or even promote antigen-specific immune responses. In oncological settings, indolent inflammatory reactions have been consistently associated with accelerated disease progression and resistance to treatment. Conversely, adaptive immune responses specific for tumor-associated antigens are generally restraining tumor development and contribute to treatment sensitivity. Here, we harnessed female C57BL/6J mice lacking key regulators of MPT-driven necrosis and necroptosis to investigate whether whole-body defects in these pathways would influence mammary carcinogenesis as driven by subcutaneous slow-release medroxyprogesterone acetate (MPA, M) pellets plus orally administered 7,12-dimethylbenz[*a*]anthracene (DMBA, D), an in vivo model that recapitulates multiple facets of the biology and immunology of human hormone receptor positive (HR^+^) breast cancer. Our data demonstrate that female mice bearing a whole-body, homozygous deletion in peptidylprolyl isomerase F (*Ppif*), which encodes a key regulator of MPT-driven necrosis commonly known as CYPD, but not female mice with systemic defects in necroptosis as imposed by the whole body-deletion homozygous of receptor-interacting serine-threonine kinase 3 (*Ripk3*) or mixed lineage kinase domain like pseudokinase (*Mlkl*), are more susceptible to M/D-driven carcinogenesis than their wild-type counterparts. These findings point to CYPD as to an oncosuppressive protein that restrains HR^+^ mammary carcinogenesis in mice, at least potentially via MPT-driven necrosis.

## Introduction

Mammalian cells are equipped with a variety of mechanisms that ensure their controlled demise in both physiological and pathological settings [1–3]. Indeed, while for a long time apoptosis was believed to be the sole cell death pathway to be genetically controlled, it is now widely accepted that mammalian cells can also undergo various regulated forms of necrosis [1–3]. These include (but are not limited to): (1) mitochondrial permeability transition (MPT)-driven necrosis, which is precipitated by peptidylprolyl isomerase F (PPIF, also known as CYPD) [4], and (2) necroptosis, which requires the kinase activity of receptor-interacting serine-threonine kinase 3 (RIPK3) as well as the ability of mixed lineage kinase domain like pseudokinase (MLKL) to form pores in the plasma membrane [5, 6]. Importantly, both these cell death subroutines can drive inflammation if not be overtly immunogenic (i.e., elicit antigen-specific immune responses associated with immunological memory) [7–9], at least in part reflecting: (1) the ability of multiple mitochondrial components to drive inflammation once released in the cytosol downstream of MPT [10], and (2) the ability of RIPK3 to engage inflammasome signaling and hence promote the maturation and release of interleukin 1 beta (IL1B) and IL18 (Ref. 6). Thus, at least a priori, pre-malignant cells undergoing MPT-driven necrosis or necroptosis as a consequence of adverse microenvironmental conditions may elicit inflammatory processes or adaptive immune responses that drive [11, 12] or restrain [8] tumor progression, respectively.

We harnessed female C57BL/6J mice bearing whole-body, homozygous deletions in *Ppif*, *Ripk3* or *Mlkl* to test whether systemic defects in MPT-driven necrosis or necroptosis influence mammary carcinogenesis as elicited by the subcutaneous implantation of slow-release medroxyprogesterone acetate (MPA, M) pellets coupled with the oral administration of 7,12-dimethylbenz[*a*]anthracene (DMBA, D) [13–15]. We focused on these specific genes not only because they are mechanistically involved in MPT-driven necrosis (*Ppif*) [4] and necroptosis (*Ripk3*, *Mlkl*) [5, 6], but also because their whole-body deletion fails to affect survival at birth and fertility in mice. Moreover, we deliberately chose this mouse model of HR^+^ mammary carcinogenesis because of the unique immunobiological resemblance to its human counterpart. Besides sharing transcriptional features with human HR^+^HER2^−^ breast cancer [13], M/D-driven mammary carcinomas established in immunocompetent female C57BL/6J mice are indeed poorly infiltrated by immune cells at baseline, and hence are poorly responsive to immune checkpoint inhibitors specific for PD-1 [13], but exquisitely sensitive to CDK4/6 inhibitors [14], similar to their human counterparts [16–18]. Moreover, M/D-driven mammary carcinogenesis appears to be susceptible to risk factors similarly increasing the propensity of postmenopausal women to develop HR^+^ breast cancer, such as obesity [13, 19]. Finally, M/D-driven mammary carcinomas not only fail to express erb-b2 receptor tyrosine kinase 2 (ERBB2, best known as HER2), but most often also preserve estrogen receptor 1 (ESR1) and progesterone receptor (PGR) expression throughout the oncogenic process [13], hence exhibiting fundamental differences to other mouse models of breast cancer expressing HRs such as MMTV-PyMT mice. Indeed, the latter robustly express HER2 and tend to lose HR expression by the time mice are randomized to treatment or tumors are collected to generate cell lines [20–22], *de facto* modeling another type (HER2^+^) of breast cancer.

We found that female *Ppif*^*−/−*^ mice, but not their *Ripk3*^*−/−*^ or *Mlkl*^*−/−*^ counterparts, develop M/D-driven mammary carcinomas with a shorter delay than wild-type (WT) mice at both primary and secondary disease sites, resulting in reduced overall survival despite a comparable growth of established tumors. These findings indicate that CYPD restrains the initial steps of HR^+^ mammary carcinogenesis in mice, at least potentially through its fundamental role in the control of MPT-driven necrosis.

## Results

### *Ppif* restrains primary M/D-driven mammary carcinogenesis

**Fig. 1:**
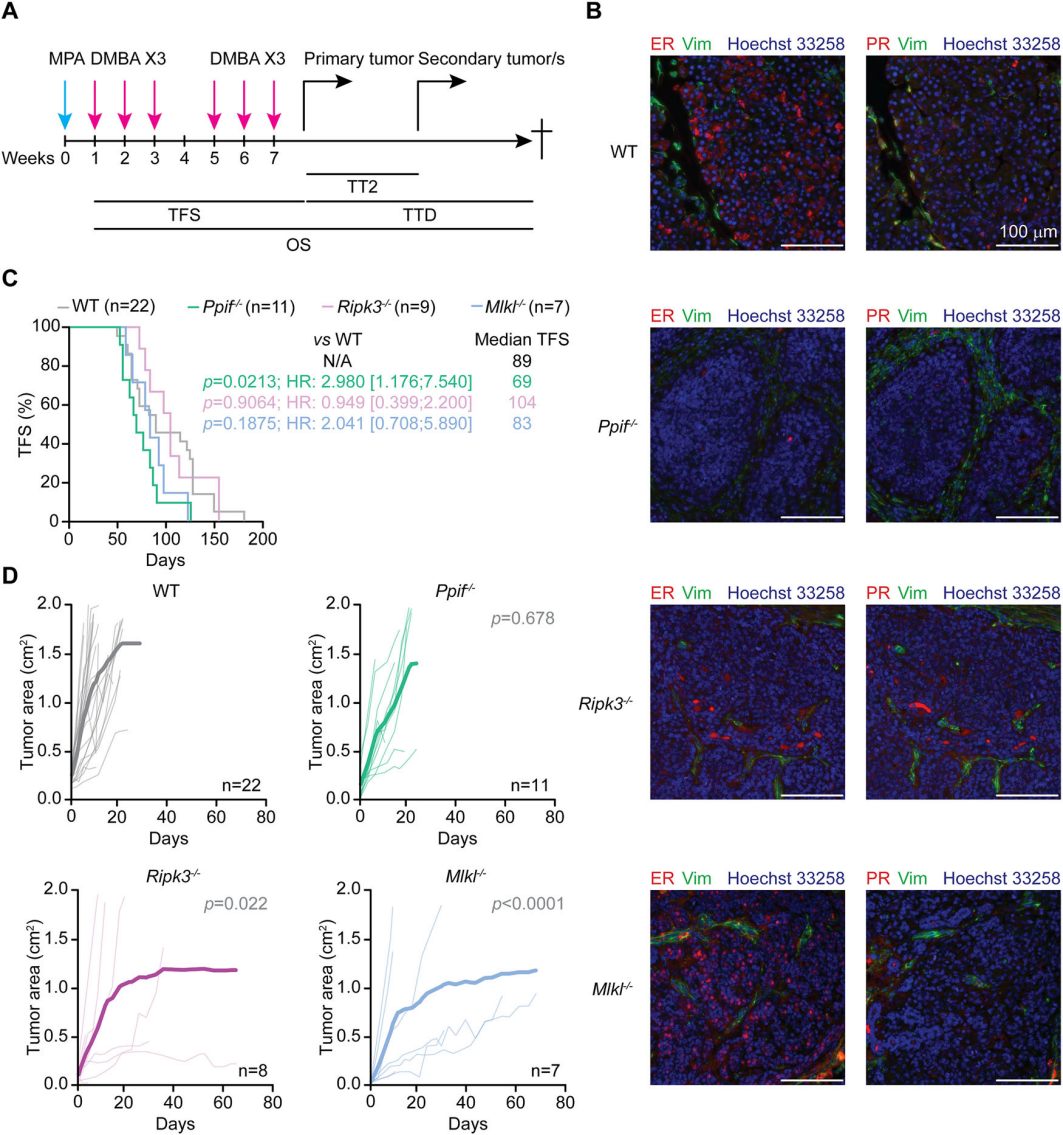
*Ppif* restrains primary M/D-driven mammary carcinogenesis. Wild-type (WT) *Ppif*^−/−^, *Ripk3*^*−/*^^−^ or *Mlkl*^*−/*^^−^ female C57BL/6J mice were subjected M/D-driven carcinogenesis, then assessed for tumor-free survival (TFS), time to secondary disease (TT2), time to death (TTD) and overall survival (OS), as well as routinely monitored for tumor growth at primary and secondary disease sites (**A**). Representative images of M/D carcinomas collected from WT, *Ppif*^−^^*/−*^, *Ripk3*^−/−^ or *Mlkl*^−/−^ mice at euthanasia and co-immunostained for ER/VIM or PR/VIM and Hoechst 33258 for nuclear counterstaining (**B**), as well as TFS (**C**) and tumor growth at primary disease site (**D**) are reported. In (**C**) median TFS, Mantel–Haenszel hazard ratio (HR) with 95% confidence interval (CI), group size (*n*) and *p* values (Log-rank, compared to WT mice) are indicated. In (**D**) both individual and average tumor growth are illustrated, with group size (*n*) and *p* values (2-way ANOVA, compared to WT mice) reported.

To elucidate the impact of systemic defects in MPT-driven necrosis and necroptosis on HR^+^ mammary carcinogenesis, we subjected female WT, *Ppif*^*−/−*^, *Ripk3*^*−/−*^, and *Mlkl*^*−/−*^ C57BL/6J mice of 6–9 weeks of age to M/D-driven mammary carcinogenesis according to established procedures [13, 23], and monitored them for tumor-free survival (TFS), as well as for a number of other parameters defining disease progression (Fig. 1A). In line with previous findings from us and others [13, 24], female WT mice developed M/D-driven mammary carcinomas expressing estrogen receptor 1 (ESR1, best known as ER) and progesterone receptor (PGR, best known as PR), but not vimentin (VIM)—*de facto* exhibiting a luminal phenotype—with complete penetrance and a median latency of 89 days from the 1st DMBA gavage (Fig. 1B, C). Neither the *Ripk3*^*−/−*^ nor the *Mlkl*^*−/−*^ genotype significantly influenced tumor penetrance (data not shown), phenotype (Fig. 1B) or latency (median TFS: 104 days and 83 days, respectively; *p* value: 0.9064 and 0.1875, respectively) in this setting (Fig. 1C). Conversely, while *Ppif*^*−/−*^ mice also developed M/D-driven mammary tumors with complete penetrance (data not shown), these lesions expressed limited ER levels (Fig. 1B) and emerged with significantly accelerated kinetic as compared to their WT counterparts (median TFS: 69 days; *p* value: 0.0213) (Fig. 1C). However, the growth of first detectable (primary) M/D-driven carcinomas, as monitored from tumor detection with a common caliper, did not differ between *Ppif*^*−/−*^ and WT mice (*p* value: 0. 678), while it was slightly (but significantly) reduced in their *Ripk3*^*−/−*^ and *Mlkl*^*−/−*^ genotype (*p* value: 0.022 and <0.0001, respectively) (Fig. 1D).

These findings demonstrate that the whole-body homozygous deletion of *Ppif* shortens the latency for M/D-driven mammary carcinomas to become detectable in the absence of overt alterations in tumor growth rate.

### Lack of *Ppif* promotes secondary M/D-driven mammary carcinogenesis

**Fig. 2:**
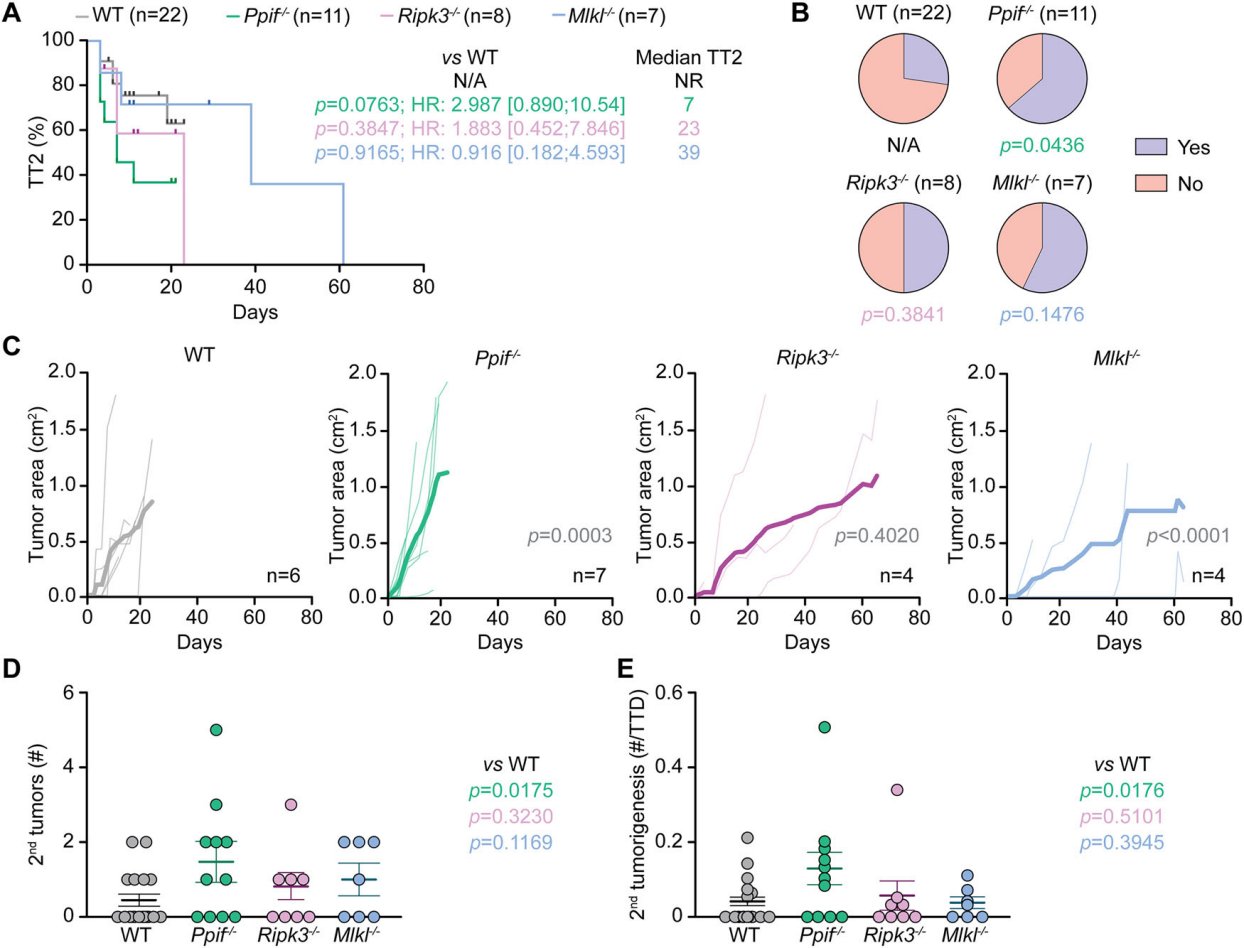
Lack of *Ppif* promotes secondary M/D-driven mammary carcinogenesis. Wild-type (WT) *Ppif*^−/−^, *Ripk3*^*−/*^^−^ or *Mlkl*^*−/−*^ female C57BL/6J mice were subjected M/D-driven carcinogenesis and analyzed as illustrated in Fig. 1A. Time to secondary disease (TT2) (**A**), percentage of mice developing secondary lesions (**B**), secondary tumor growth (**C**), number of secondary tumors per mouse (**D**) and number of secondary tumors per mouse normalized to time to death (TTD) (**E**) are reported. In (**A**), median TT2, Mantel–Haenszel hazard ratio (HR) with 95% confidence interval (CI), group size (*n*) and *p* values (Log-rank) are indicated. Mice succumbing to primary disease without developing a secondary tumor were censored from the analysis. In (**B**), group size (*n*) and *p* values (Fisher’s exact test, compared to WT mice) are indicated. In (**C**) both individual and average tumor growth are illustrated, with group size (*n*) and *p* values (2-way ANOVA, compared to WT mice) reported. In (**D** and **E**) results are reported as means ± SEM and individual data points, *p* values (Kruskal–Wallis, compared to WT mice) are indicated. NR not reached.

M/D-driven oncogenesis proceeds beyond the formation of detectable primary tumors, resulting in the appearance of extra (secondary) lesions that contribute to cumulative tumor burden and hence to the definition of humane endpoint [15]. To understand the impact of genetic alterations in key molecular regulators of MPT-driven necrosis and necroptosis, we thus assessed time to secondary oncogenesis (TT2), defined as the number of days elapsing between the detection of the primary M/D-driven tumor and any extra mammary lesions emerging thereafter. Most often, WT mice had to be euthanatized because of the uncontrolled growth of primary M/D-driven tumors before developing a secondary neoplasm, hence failing to reach median TT2 (Fig. 2A). Indeed, only 6/22 (~ 27.3%) mice in this group developed at least one secondary tumor by the time euthanasia was required owing to global disease burden (Fig. 2B). *Ripk3*^*−/−*^ and *Mlkl*^−^^*/−*^ mice exhibited a median TT2 of 23 and 39 days, respectively, which was not significantly different compared to WT mice (*p* value: 0.3847 and 0.9165, respectively) (Fig. 2A). Accordingly, 4/8 (50%) *Ripk3*^−/−^ mice and 4/7 (~ 57.1%) *Mlkl*^*−/*^^−^ mice developed at least one secondary lesion before global tumor burden reached ethical endpoint, which failed to differ in a statistically significant manner from WT mice (*p* value: 0.3841 and 0.1476, respectively) (Fig. 2B). Conversely, secondary M/D-driven tumorigenesis exhibited a strong (although sub-significant) trend towards acceleration in *Ppif*^*−/−*^ mice (median TT2: 7 days; *p* value: 0.0763), and these animals developed at least one secondary tumor in 7/11 cases (~ 63.6%, *p* value: 0.0436) (Fig. 2A, B).

Of note, secondary M/D-driven tumors failed to exhibit differences in growth pattern when *Ripk3*^*−/−*^ mice were compared to their WT counterparts in this respect (*p* value: 0.4020) (Fig. 2C). Conversely, while secondary M/D-driven carcinomas evolving in *Mlkl*^*-/-*^ mice grew less rapidly compared to the same tumors progressing in WT mice (*p* value: <0.0001), the contrary was true for secondary M/D-driven tumors developing in *Ppif*^−/−^ mice (*p* value: 0.0003) (Fig. 2C). Finally, *Ripk3*^*−/−*^ mice and *Mlkl*^−^^*/−*^ mice did not differ from WT mice with respect to the number of secondary M/D-driven tumors per mouse (*p* value: 0.3230 and 0.1169, respectively), even when this parameter was normalized for mouse survival (*p* value: 0.5101 and 0.3945, respectively) (Fig. 2D, E). On the contrary, *Ppif*^*−/−*^ mice subjected to M/D-driven carcinogenesis accumulated—in average—an increased amount of secondary lesions per mouse as compared to WT mice, not only as an absolute measurement (*p* value: 0.0175), but also upon accounting for differential survival (*p* value: 0.0176) (Fig. 2D, E).

Collectively, these data demonstrate that the whole-body deletion of *Ppif* accelerates HR^+^ carcinogenesis as driven in C57BL/6J mice by MPA and DMBA not only at primary, but also at secondary, disease sites.

### *Ppif* inhibits natural disease progression in M/D-driven mammary carcinomas

**Fig. 3:**
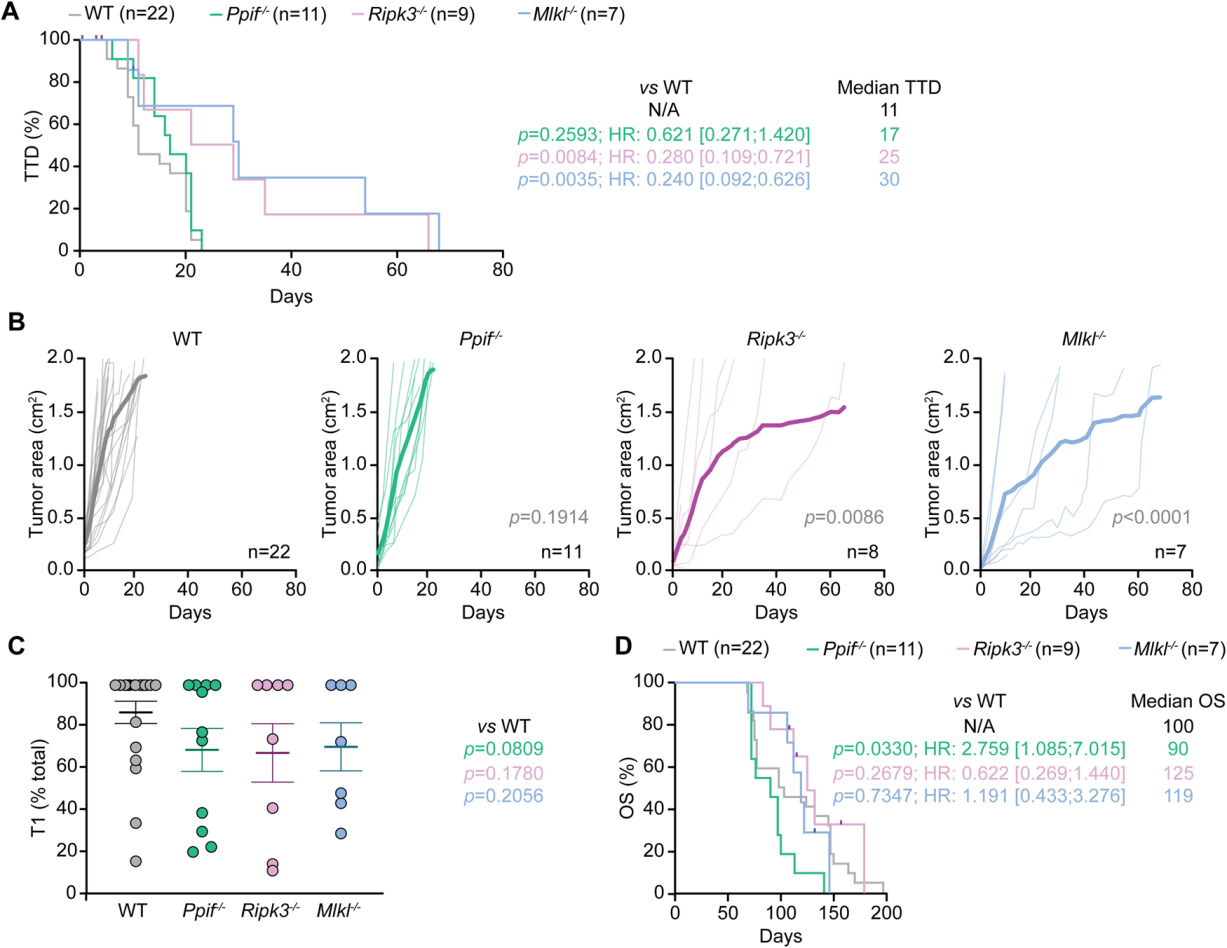
*Ppif* inhibits natural disease progression in M/D-driven mammary carcinomas. Wild-type (WT) *Ppif*^−/−^, *Ripk3*^−^^*/−*^ or *Mlkl*^−^^*/−*^ female C57BL/6J mice were subjected M/D-driven carcinogenesis and analyzed as illustrated in Fig. 1A. Time to death (TTD) (**A**), cumulative tumor growth (**B**), relative contribution of primary tumors (T1) to overall disease burden at euthanasia (**C**) and overall survival (OS) (**D**) are illustrated. In (**A** and **D**) median values, Mantel–Haenszel hazard ratio (HR) with 95% confidence interval (CI), group size (*n*), and *p* values (Log-rank) are indicated. Mice succumbing to causes other than euthanasia owing to global disease burden were censored from the analysis. In (**B**) both individual and average tumor growth are illustrated, with group size (n) and *p* values (2-way ANOVA, compared to WT mice) reported. In (**C**) results are reported as means ± SEM and individual data points, *p* values (Kruskal–Wallis, compared to WT mice) are indicated.

Despite the early appearance of primary and secondary M/D-driven tumors as well as the accelerated tumor growth at secondary disease sites as documented in female *Ppif*^−^^*/−*^ mice (Figs. 1B and 2A–C), these animals exhibited a median time to death (TTD), defined as the number of days elapsing between the detection of the first malignant lesion and ethical endpoint as dictated by cumulative tumor burden, of 17 days, which was not significantly different from that of WT mice (median TTD: 11 days, *p* value: 0.2593) (Fig. 3A), potentially owing to a slight (although sub-significant) deceleration in primary tumor growth (Fig. 1C). In line with this notion, the *Ripk3*^*−/*^^−^ and even more so the *Mlkl*^*−/*^^−^ genotype were associated with an extension in TTD (median TTD: 25 and 30 days, respectively; *p* value: 0.0084 and 0.0035, respectively) (Fig. 3A), largely reflecting the reduced speed of tumor progression at primary disease sites (Fig. 1B) in the context of limited alterations in TT2 and secondary tumor growth (Fig. 2A–C). Consistent with this notion, while the growth of all detectable M/D-driven tumors failed to differ between WT and *Ppif*^−/−^ mice (*p* value: 0.1914), both the *Ripk3*^*−/−*^ and the *Mlkl*^*−/*^^−^ genotype (*p* value: 0.0086 and <0.0001, respectively) were associated with significant reduction in global disease progression (Fig. 3B).

Of note, the relative contribution of primary disease to global tumor burden as a determinant of ethical endpoint was not affected by the whole-body deletion of *Ripk3* (*p* value: 0.1780) or *Mlkl* (*p* value: 0.2056) (Fig. 3C). Conversely, the *Ppif*^*−/−*^ genotype tended to be associated (although in a sub-significant manner) with a decreased relative contribution of primary over secondary tumors to global disease burden at ethical endpoint (*p* value: 0.0809) (Fig. 3C). Moreover, while both the *Ripk3*^−^^*/−*^ and the *Mlkl*^*−/*^^−^ genotype failed to influence the overall survival (OS) of female mice subjected to M/D-driven carcinogenesis (median OS: 125 and 119 days, respectively; *p* value: 0.2679 and 0.7347, respectively), the whole-body deletion of *Ppif* significantly shortened it (median OS: 90 days; *p* value: 0.0330), with WT animals exhibiting a median OS of 100 days (Fig. 3D).

Taken together, these data indicate that CYPD restrains the natural progression of HR^+^ mammary carcinogenesis in female C57BL/6J mice by interfering with early stages of tumorigenesis.

## Discussion

In summary, our data indicate that CYPD—a fundamental regulator of MPT-driven necrosis [25, 26]—mediates oncosuppressive effects in an immunocompetent mouse model of HR^+^ mammary oncogenesis driven by the systemic administration of a chemical carcinogen, i.e., DMBA, in the context of supraphysiological PR signaling, as elicited by slow-release MPA pellets [13–15]. As introduced above, this is a uniquely translational model of HR^+^HER2^−^ oncogenesis, as it recapitulates a number of biological, immunological and therapeutic aspects of its human counterpart [13–15], hence standing out as a preferential platform for immuno-oncology studies of this specific variant of breast cancer [27, 28]. Moreover, our findings are fully in line with the well-recognized oncosuppressive role of regulated cell death (RCD) in many of its variants [29–31], largely (but perhaps not exclusively) reflecting the evolutionary advantage provided to a multicellular organism by signal transduction cascades that coordinate the demise of individual cells bearing excessive macromolecular damage (hence being unable to perform their physiological functions or even at increased risk of malignant transformation) in the context of adequate immunological responses [32, 33].

CYPD has been shown to mediate various functions that may or may not involve MPT regulation but definitely do not culminate with MPT-driven necrosis, including a paradoxical cytoprotective function in senescent cells, as well as a metabolic activity in hematopoietic precursors [34–36]. Thus, it remains possible that the ability of CYPD to suppress HR^+^ mammary carcinogenesis in female C57BL/6J mice may be unrelated to RCD via MPT-driven necrosis, but may instead involve complex systemic effects originating in compartments other than (pre-)malignant niches. This is particularly challenging to formally establish with additional genetic approaches [37], as most (if not all) proteins that reportedly form or interact with—hence regulating—the supramolecular complex responsible for MPT, which is commonly known the permeability transition pore complex: (1) exhibit considerable genetic and/or functional redundancy, considerably complicating the implementation of successful knockout strategies in vivo; [38, 39] (2) are critical components of the molecular machinery that ensure mitochondrial ATP synthesis, *de facto* being strictly required for survival; [40–42] and (3) at least in some cases, have been conclusively shown to be dispensable for MPT [25, 43, 44]. Along similar lines, currently available pharmacological inhibitors of the MPT exhibit limited specificity [37]. As a standalone example, the pharmacological CYPD inhibitor cyclosporin A (CsA) has major CYPD-independent immunosuppressive effects by inhibiting peptidylprolyl isomerase A (best known as CYPA) in T cells [45, 46].

Intriguingly, CYPD has also been shown to contribute to normal T cell and natural killer (NK) cell functions, at least in preclinical models of infection [47, 48], raising the possibility that accelerated MPA/DMBA-driven mammary carcinogenesis as observed in *Ppif*^*−/−*^ C57BL/6J mice may result from defects in natural immunosurveillance [49]. We have previously demonstrated that MPA/DMBA-driven mammary tumors develop with an accelerated kinetic in *Rag2*^*−/−*^*Il2rg*^−^^*/*^^−^ mice (which lack T cells, B cells and NK cells), as well as in mice receiving an antibody specific for NKG2D (which depletes NK cells and a subpopulation of CD8^+^ T cells), but not in *Rag2*^*−/−*^ mice (which lack T and B cells) or in mice receiving CD4- and CD8-targeting antibodies (which are depleted of T cells), globally pointing to NK cells as to central mediators of natural immunosurveillance in this model [13, 50]. Subjecting C57BL/6J mice to total body irradiation-induced myeloablation and reconstituting them with *Ppif*^−/−^ hematopoietic stem cells (and vice versa) will provide additional insights into the role of CYPD expression in radiosensitive vs radioresistant cells in MPA/DMBA-driven mammary carcinogenesis.

Despite this and other open avenues, our findings indicate that CYPD retards HR^+^ mammary carcinogenesis in immunocompetent C57BL/6J mice. Of note, CYPD has previously been shown to promote (rather than inhibit) hepatocellular carcinogenesis in mice with non-alcoholic steatohepatitis [51] as well HR^+^HER2^+^ mammary carcinogenesis as driven by the MMTV-PyMT construct [52]. Additional work is hence required to understand whether our data reflect unique immunobiological features of HR^+^ breast cancer over other breast cancer subtypes and extramammary neoplasms.

## Materials and methods

### Ethics approval and consent to participate

Animal studies were performed as per guidelines from the Guide for the Care and Use of Laboratory Animals [53] and under a protocol approved by the Institutional Animal Care and Use Committee of Weill Cornell Medical College (n° 2020-0022). No human subjects were included in this study,

### Mice and oncogenesis

Endogenous mammary carcinogenesis was initiated as previously described [13, 23]. Shortly, a 50 mg slow-release (90 days) medroxyprogesterone acetate (MPA, M) pellet (#NP-161, Innovative Research of America) was implanted subcutaneously in the interscapular area of 6–9 weeks old female C57BL/6J mice (*Mus musculus*, from Jackson). One week later, mice received 1 mg 7,12-dimethylbenz[a]anthracene (DMBA, D) in 200 µL corn oil (#C8267, Millipore Sigma) by oral gavage, a procedure that was repeated on weeks 2, 3, 5, 6, and 7 after implantation of the MPA pellet [13, 23]. Mice were routinely checked for the appearance of mammary lesions, which were monitored for growth with a common caliper. Mice were euthanatized when the cumulative surface of all neoplastic lesions (computed as the area of an ellipse: A = longest diameter X shortest diameter X π/4) reached 180–200 mm^2^ (ethical endpoint that was employed as surrogate marker for survival), or in the context of evident toxicity or distress (e.g., hunching, anorexia, tumor ulceration).

### Immunofluorescence microscopy

M/D-driven tumors collected at euthanasia (see above) were fixed in 4% paraformaldehyde in PBS, embedded in paraffin and cut into 5 μm-thick sections as per conventional procedures [13]. Upon adsorption onto charged microscope slides (#1358, Globe Scientific), sections were deparaffinized and re-hydrated by 3× incubations of 10 min each in xylenes (#534056, Millipore Sigma), followed by 2× incubations of 5 min each in 100%, 90%, 80%, and 70% ethanol. Sections were boiled for 30 min in Antigen Retrieval Buffer (1× Tris-EDTA Buffer, pH 9.0) (#ab93684, from Abcam), rinsed 4× with TBS, and incubated in 3% BSA in TBS-Tween for 30 min at RT to block non-specific binding site. Slides were incubated overnight at 4 °C with the following primary antibodies: anti-ERalpha (1:20; #MA1-80216, Thermo Fisher Scientific), anti-PR (1:20, #MA1-410; Thermo Fisher Scientific), and anti-VIM (1:200; #GTX100619, GeneTex). Slides were next rinsed 3× with TBS-Tween, followed by incubation with Goat anti-Rabbit IgG (H + L) Cross-Adsorbed Secondary Antibody, Alexa Fluor™ 488 (1:500, #A-11008, Thermo Fisher Scientific) and Donkey anti-Mouse IgG (H + L) Highly Cross-Adsorbed Secondary Antibody, Alexa Fluor™ 555 (1:500; #A31570, Thermo Fisher Scientific) for 30–60 min at RT. Slides were rinsed 3× with TBS-Tween, incubated with Vector® TrueVIEW® Autofluorescence Quenching Kit (#SP-8400, Vector Laboratories, Inc.) for 5 min at RT, and washed 1× in PBS. Finally, slides were incubated in 5 µg mL^−1^ Hoechst 33258 (#H3569, Thermo Fisher) for 10 min at RT, mounted with ProLong™ Glass Antifade Mountant (#P36984, Thermo Fisher), and imaged on a Leica DMi8 inverted fluorescence microscope operated by Leica Application Suite X, version 3.7.4.23463 (Leica Microsystems).

### Data processing and statistical analysis

The following parameters were measured or scored: (1) TFS, defined as the number of days between the 1st DMBA administration and the detection of the first malignant lesion; (2) time to secondary disease (TT2), defined as the number of days between the detection of the first malignant lesions and the detection of any other malignant lesion; (3) time to death (TTD), defined as the number of days between the detection of the first malignant lesions and ethical endpoint (see above); (4) overall survival (OS), defined as the number of days between the 1st DMBA administration and ethical endpoint (see above); (5) number of secondary tumors at euthanasia; (6) normalized number of secondary tumors at euthanasia, defined as the number of secondary tumors at euthanasia divided by TTD; (7) % of primary tumor burden (T1) at euthanasia, defined as follows: T1 (%) = 100 × surface area of the primary tumor/surface area of all tumors; (8) primary tumor growth; (9) secondary tumor growth; (10) cumulative tumor growth. Prism v. 10.2.3 (GraphPad) and Excel 2021 (Microsoft) were used for data processing, plotting, and statistical analysis. Illustrator 2025 (Adobe) was used for figure preparation.

One-way ANOVA plus Geisser-Greenhouse correction and Fisher’s LSD were applied to assess statistical significance in comparisons involving numerical data (which were normally distributed and exhibited comparable variance). Incidence of secondary oncogenesis was assessed for statistical significance by Fisher’s exact test. Growth curves were assessed for statistical significance by two-way ANOVA plus Geisser-Greenhouse correction. TFS, TT2, TTD, and OS curves were assessed for statistical significance by Log-rank (Mantel-Cox) and Mantel–Haenszel tests. Whenever relevant, number of mice per group, hazard ratio (HR) plus 95% confidence interval (CI) and *p* values are reported.

Groups of 10 mice per genotype were planned as per our previous experience with oncogenesis in this model [13, 23]. Whenever possible, larger groups were used to improve statistical power. As pre-established criteria, mice requiring euthanasia for oncogenesis-unrelated causes prior to tumor development were completely excluded from this study. Moreover, mice succumbing to endpoint-unrelated causes were censored from statistical assessments on TT2, TTD, and OS curves. As per the nature of this study (purely prophylactic), no randomization was implemented, and all experimental assessments were performed in an unblinded manner.

## Data Availability

All data supporting these findings are available from the corresponding authors upon reasonable request.
